# Correction to “Predictive Value of Clinical and Demographic Variables in Martorell Ulcers: An Analysis Based on Case Reports”

**DOI:** 10.1111/iwj.70768

**Published:** 2025-10-01

F. I. de la Rosa‐Negrón, M. Á. Castaño‐López, and F. Navarro‐Roldán, “Predictive Value of Clinical and Demographic Variables in Martorell Ulcers: An Analysis Based on Case Reports” *International Wound Journal* 22, no. 8 (2025): e70721, https://doi.org/10.1111/iwj.70721.

The epidemiological data at the end of the first paragraph of the Introduction is supported by reference no. 21 (Liroz‐Imaz A., García‐Montero A., Gombau‐Baldrich Y., and Guinot‐Bachero J., “Martorell Hypertensive Ischemic Ulcer: A Pain Perspective Approach,” *Gerokomos* 33, 3 (2022): 146–151).

During the implementation of the corrections suggested by the reviewers, this citation was inadvertently omitted.

We apologize for this error.

